# Ethnic density and area deprivation: Neighbourhood effects on Māori health and racial discrimination in Aotearoa/New Zealand

**DOI:** 10.1016/j.socscimed.2013.04.007

**Published:** 2013-07

**Authors:** Laia Bécares, Donna Cormack, Ricci Harris

**Affiliations:** a Centre on Dynamics of Ethnicity, University of Manchester, Oxford Road, Manchester M13 9PL, UK; b Te Rōpū Rangahau Hauora a Eru Pōmare, University of Otago, New Zealand

**Keywords:** Ethnic density, Racial discrimination, Māori, Self-rated health, Common mental disorders, Neighbourhood, New Zealand

## Abstract

Some studies suggest that ethnic minority people are healthier when they live in areas with a higher concentration of people from their own ethnic group, a so-called ethnic density effect. To date, no studies have examined the ethnic density effect among indigenous peoples, for whom connections to land, patterns of settlement, and drivers of residential location may differ from ethnic minority populations.

The present study analysed the Māori sample from the 2006/07 New Zealand Health Survey to examine the association between increased Māori ethnic density, area deprivation, health, and experiences of racial discrimination. Results of multilevel regressions showed that an increase in Māori ethnic density was associated with decreased odds of reporting poor self-rated health, doctor-diagnosed common mental disorders, and experienced racial discrimination. These associations were strengthened after adjusting for area deprivation, which was consistently associated with increased odds of reporting poor health and reports of racial discrimination. Our findings show that whereas ethnic density is protective of the health and exposure to racial discrimination of Māori, this effect is concealed by the detrimental effect of area deprivation, signalling that the benefits of ethnic density must be interpreted within the current socio-political context. This includes the institutional structures and racist practices that have created existing health and socioeconomic inequities in the first place, and maintain the unequal distribution of concentrated poverty in areas of high Māori density. Addressing poverty and the inequitable distribution of socioeconomic resources by ethnicity and place in New Zealand is vital to improving health and reducing inequalities. Given the racialised nature of access to goods, services, and opportunities within New Zealand society, this also requires a strong commitment to eliminating racism. Such commitment and action will allow the benefits potentially flowing from strong communities to be fully realised.

## Highlights

• Māori ethnic density is associated with improved Māori health and reduced racial discrimination.
• Area deprivation is strongly associated with poorer health and increased racial discrimination.
• The benefits of Māori ethnic density are concealed by the detrimental effect of area deprivation.

## Introduction

Māori are the indigenous people of New Zealand (NZ), comprising 15% of the total population (Statistics New Zealand, 2007). Similar to other indigenous populations, historical insults and ongoing colonisation since the nineteenth century has resulted in the large-scale dispossession and marginalisation of Māori from their social, cultural and economic resources, including land (Reid & Robson, 2007; United Nations, 2008). In contemporary NZ society, the negative effects of colonisation for Māori are manifest in stark inequities in health and other social outcomes compared with the non-indigenous population. For example, Māori life expectancy at birth is approximately 8.3 years less than non-Māori (Tobias et al., 2009), and morbidity differences have been reported across several health indicators, including most major chronic and infectious diseases (Ministry of Health, 2010). Māori are disproportionately impacted by the unequal distribution of social determinants of health, including socioeconomic resources and experiences of racial discrimination (Harris et al., 2006a, 2006b, 2012; Robson, Cormack, & Cram, 2007). Historical and more recent processes of colonisation, including asset loss, land alienation and rapid urbanisation (Te Puni Kokiri, 2000) have contributed to the concentration of Māori in particular residential areas, characterised by higher levels of deprivation. More than half of Māori live in areas considered to be among the most deprived in the country (Ministry of Health, 2010), a factor likely to contribute to health inequalities, given the documented association between area deprivation and poor health (Ministry of Health, 2012; Pickett & Pearl, 2001; Riva, Gauvin, & Barnett, 2007; Stevenson, Pearce, Blakely, Ivory, & Witten, 2009).

Despite the international evidence on the detrimental association between area deprivation and health, areas with higher concentrations of ethnic minorities have been found to exhibit health protective effects for ethnic minority residents, relative to areas with lower residential concentrations of ethnic minority people. Theoretical discourses on this ‘ethnic density effect’ hypothesise that positive health outcomes may be attributed to the buffering effect that enhanced social cohesion, mutual social support and a stronger sense of community provide against the direct or indirect consequences of discrimination and racial harassment (Bécares, Nazroo, & Stafford, 2009; Faris & Dunham, 1939; Halpern & Nazroo, 2000). In support of this hypothesis, several studies have reported reduced experiences of racial harassment by ethnic minority residents in areas of higher ethnic density (Bécares et al., 2009; Das-Munshi, Bécares, Stansfeld, & Prince, 2010).

However, the majority of ethnic density studies have been conducted in the US and in the UK (Bécares et al., 2012; Shaw et al., 2012), and the findings cannot necessarily be generalised to migrant and ethnic minority populations in other national contexts, or to indigenous populations in their homelands. Explorations of the ethnic density effect in NZ are particularly poignant given Māori indigenous rights and connections to their land, which have to some extent been more broadly recognised, if not realised (United Nations, 2008). The United Nations Declaration on the Rights of Indigenous Peoples, while recognising the diversity and specificity of histories and contexts, acknowledges the relationships between indigenous populations and their environments, and their right to preserve connections with land and with cultural and historical sites (United Nations, 2008). Dispossession, unlawful transfer and forced removal from land are experiences common to many indigenous communities (Fleras & Spoonley, 1999; King, Smith, & Gracey, 2009; United Nations, 2008), as are acts of resistance and efforts to retain and regain land (Churchill, 1995). Colonial policies challenged Māori cultural, political and tribal relationships with the land, imposed new administrative arrangements over existing Māori structures (Fleras & Spoonley, 1999; Smith, 1996), and encouraged the movement of Māori away from rural to urban areas for employment and resettlement (Hill, 2012; Pearson, 2001; Smith, 1996; Walker, 1996). Although there is increased acknowledgement of links between place and health for indigenous populations (Durie, 2003; King et al., 2009), no studies have yet examined the ethnic density effect among indigenous peoples, for whom connections to land, patterns of settlement, and drivers of residential location may differ from non-indigenous populations in the same territory. For Māori, this includes historical and cultural relationships, and political policies governing land use and ownership, and access to employment and other economic and social resources.

This study aims to investigate the ethnic density effect among Māori, hypothesising that an increase in Māori ethnic density will be associated with better health outcomes. Given the high prevalence of experienced racial discrimination among Māori, and the documented association between experienced racial discrimination and poor health (Harris et al., 2006a, 2006b, 2012), a second aim of the present study is to examine whether Māori ethnic density is associated with experiences of racial discrimination. We hypothesise that, as has been documented in UK studies (Bécares et al., 2009; Das-Munshi et al., 2010), an increase in Māori ethnic density will be associated with decreased reports of racial discrimination among Māori.

We also aim to explore whether the associations between Māori ethnic density and health, and Māori ethnic density and racial discrimination, persist once the effects of area deprivation are taken into account; and to examine the relative contribution of ethnic density and deprivation to health and racial discrimination among Māori in NZ.

## Methods

### Study population

This study uses the Māori sample from the 2006/07 New Zealand Health Survey (NZHS). The NZHS is a national survey conducted at regular intervals (now continuously) by the New Zealand Ministry of Health, with the aim of obtaining detailed information on health status, health service utilisation, and health risk factors, including experiences of racial discrimination, among the usually resident New Zealand population living in private dwellings (Ministry of Health, 2008). Data for the 2006/07 NZHS were collected between October 2006 and November 2007 using a multi-stage, stratified, probability-proportional-to-size (PPS) sampling design. A total of 1385 primary sampling units (called *meshblocks*: small areas of about 100 people) were selected from across the country. Within each selected meshblock, all dwellings were enumerated and then two separate random samples of dwellings were selected: a ‘core’ sample in which all adults were eligible, and an extra ‘screened’ sample from which only Māori, Pacific or Asian adults were eligible. In total, across all the selected meshblocks, 14,571 households were selected for the core sample and 20,998 households were selected for the screened sample. Within each selected household in the core sample, all eligible adults (aged 15 years and older) were identified and one was randomly selected as the respondent. Within each selected household in the screened sample, all Māori, Pacific or Asian adults (aged 15 years and older) were identified and one was randomly selected as the respondent. No interview was conducted in households in the screened sample if there were no household members who identified as Māori, Pacific or Asian.

A total of 12,488 face-to-face interviews (67.9% weighted response rate) were conducted in English by trained interviewers. A total of 3160 interviews were conducted with Māori respondents (67.5% Māori weighted response rate).

### Outcome variables: health

The association between Māori ethnic density and health was examined using three different health outcomes: overall self-rated health, doctor-diagnosed common mental disorders, and psychological distress.

Overall self-rated health has been previously used in studies of ethnic density (Bécares et al., 2012), and has been shown to be a valid indicator of health status. Reports of poor health have been associated with higher mortality, psychological distress, and poor functioning (Idler & Benyamini, 1997; Krause & Jay, 1994). In the NZHS, respondents were asked to rate their health on a 5-point Likert scale ranging from excellent to poor. Responses were dichotomised into excellent, very good, and good, or fair, and poor.

Doctor-diagnosed common mental disorders were assessed by asking respondents whether they had been diagnosed with any of a series of conditions that had lasted, or were expected to last, for more than six months, including depression, bipolar disorder, anxiety disorder, eating disorder, alcohol use disorder, drug use disorder, schizophrenia, or other mental health conditions. We considered a doctor-diagnosed common mental disorder to be present if respondents answered ‘yes’ to one or more of these conditions.

Psychological distress was measured with the Kessler-10 item scale (Kessler et al., 2002, 2003), which consists of ten questions on negative emotional states experienced in the four weeks prior to interview. The K10 generates a continuous score ranging from zero to 40, with higher scores indicating increased psychological distress.

### Outcome variables: racial discrimination

Experiences of racial discrimination were measured with a set of variables that have been previously used to assess the prevalence of experienced racism and the association between racism and health among Māori (and other New Zealanders) in NZ (Harris et al., 2006a, 2006b, 2012). Respondents were asked whether they had ever ‘been a victim of an ethnically motivated attack (verbal or physical abuse to the person or property),’ ‘been treated unfairly (for example, kept waiting or treated differently) by a health professional because of your ethnicity,’ ‘been treated unfairly at work or been refused a job because of your ethnicity,’ and ‘been treated unfairly when renting or buying housing because of your ethnicity.’ Response categories were ‘yes, within the past 12 months,’ ‘yes, more than 12 months ago,’ ‘no’ and were dichotomised into 0: never, and 1: yes, ever. We created three summary measures that assessed whether respondents had ever experienced: an ethnically motivated personal attack (combining positive reports of either verbal or physical lifetime attacks); any unfair treatment due to their ethnicity (combining positive reports of unfair treatment by either a health professional, in the job sector, or in gaining housing); and any racial discrimination (measuring a positive response to any of the variables asking about ethnically motivated personal attack or unfair treatment).

### Predictor variables: individual-level measures

Māori ethnicity was assessed using a self-report variable as used in the 2006 NZ Census, which allows respondents to self-identify with one or more ethnic groups. Respondents were classified as Māori if they identified themselves as Māori either alone or in combination with other ethnic groups.

Socioeconomic factors considered to be confounders at the individual-level were work status (employed, not employed but seeking work, or not employed and not seeking work); highest educational qualification (no secondary school, some secondary school, post-secondary school qualifications); and equivalised household income (equivalised for household size and composition using the modified Jensen scale; Jensen, 1988).

### Predictor variables: area-level measures

Māori ethnic density and area deprivation were anonymously geocoded by the data holder (the New Zealand Ministry of Health), using the 2006 NZ Census. Area boundaries were defined using Census Area Units (CAUs) because they provide the most appropriate geographical approximation to the construct of neighbourhood, and are commonly used to study neighbourhood effects in New Zealand (see for example Blakely et al., 2006; Brown, Guy, & Broad, 2005; Ivory, Collings, Blakely, & Dew, 2011). CAUs are areas defined for statistical purposes by Statistics New Zealand, and at the time of the 2006 Census there were 1927 CAUs with an average of 2200 residents or 760 households in NZ (Statistics New Zealand, 2006). CAUs correspond reasonably closely to suburbs in urban areas and communities in rural areas and have borders based on locally recognisable communities (Statistics New Zealand, 2006).

Māori ethnic density was calculated by dividing the number of Māori residents in each CAU at the 2006 NZ Census, by the total population in that CAU.

Tests for departure from linearity in the association between ethnic density, health and racism were performed with likelihood ratio tests. Results suggested that these associations were linear, so we analysed ethnic density as a continuous variable. To aid in interpretation, and because a 1% change is not large enough to be relevant, we divided the original ethnic density variable by 10 so we could estimate the association with health and experienced racism for every 10 percentage point increase in Māori ethnic density.

Neighbourhood deprivation was assessed using the New Zealand Deprivation Index 2006 (NZDep06; Salmond, Crampton, & Atkinson, 2007), which indicates the level of deprivation of the CAU where the respondent lives relative to the whole country. NZDep06 is the first principal component derived from a principal components analysis of nine variables from the 2006 NZ Census, each of which is expressed as the age standardised proportion of residents with the characteristic in question. The nine variables are income, welfare receipt, home ownership, family structure, unemployment, qualifications, overcrowding, telephone access and car access. The NZDep06 score was categorised into quintiles, with quintile one representing the least deprived 20% of CAUs of New Zealand, and quintile five the most deprived 20% CAUs.

### Statistical analysis

The 3160 Māori respondents in the 2006/07 NZHS were clustered within 864 CAUs. To account for the hierarchical nature of the NZHS, where individuals (level 1) are nested within neighbourhoods (level 2), data were analysed using multilevel modelling, which corrected for nonindependence of observations due to geographic clustering. Random effects multilevel linear regression models were conducted to explore the association between ethnic density and psychological distress. The associations between ethnic density and self-rated health and doctor-diagnosed mental disorders, and ethnic density and experienced racial discrimination, were examined using random effects multilevel logistic regressions.

To explore the association between ethnic density and health, and ethnic density and racial discrimination, and in order to model the relative contribution of ethnic density and area deprivation to each dependent variable, we fitted regression models in three sequential steps. Model 1 included Māori ethnic density, age, and sex. Model 2 also included area deprivation; and Model 3 additionally adjusted for individual-level socioeconomic position. This allowed us to examine the crude association between Māori ethnic density and health, and Māori ethnic density and experienced racial discrimination in Model 1, and examine, upon adjustment for area deprivation in Model 2, the independent contribution that ethnic density and area deprivation have on either health or racial discrimination, as well as the role of area deprivation on concealing ethnic density effects. Additionally adjusting for individual-level socioeconomic factors in Model 3 further allowed us to understand the contribution of both ethnic density and area deprivation on health and experienced racial discrimination, independent of individual-level socioeconomic position.

We conducted both weighted and unweighted regression models to examine whether the parameter estimates of the two sets of regression models differed, and applied the DuMouchel–Duncan (1983) *F* test to test the significance of the impact of sampling weights on estimation results (DuMouchel & Duncan, 1983). We found that the weighted and unweighted estimates were not significantly different, and so we estimated all regression models without the use of sampling weights. When sampling is not based on any of the dependent variables, and sampling weights are a function of independent variables included in multivariate analyses (i.e., age, sex, ethnicity, socioeconomic status), unweighted regression estimates are preferred because they are unbiased, consistent, and tend to have smaller standard errors than weighted regression estimates (Winship & Radbill, 1994).

Descriptive statistics were weighted to account for non-response of eligible participants and the unequal probability of being sampled. All analyses were conducted in Stata 11 (StataCorp, 2009).

## Results

Table 1 presents the weighted prevalence of health outcomes and experienced racial discrimination among Māori. 13% reported their general health to be fair or poor and 14% reported a doctor-diagnosed common mental disorder. The mean Kessler-10 score was 4.6 (SD: 5.6).

Almost a third of Māori reported any experience of racial discrimination, 24% reported experiencing an ethnically motivated personal attack, and 13% reported any unfair treatment.

Māori ethnic density ranged from 2 to 86%, with a mean of 23% (see Table 1). Forty percent of Māori lived in the most deprived quintile of small areas in the country (Q5), and only 9% lived in the least deprived quintile (Q1). A positive, moderate correlation was found between area deprivation and Māori ethnic density (*r* = 0.6297).

### Māori ethnic density and health

In the crude models, an increase in Māori ethnic density was not associated with any of the three measures of health (Table 2, Model 1). Upon adjustment for area deprivation in Model 2, point estimates between increased Māori ethnic density and reports of poor self-rated health and psychological distress changed direction, becoming protective, and in the case of self-rated health, statistically significant. Effect sizes strengthened in the protective associations between increased Māori ethnic density and decreased reports of doctor-diagnosed common mental disorders.

In the fully adjusted models (Model 3), increased Māori ethnic density was found to be significantly associated with decreased odds of reporting poor self-rated health (O.R. for a 10% increase in Māori ethnic density: 0.91; 95% C.I.: 0.84–0.98) and doctor-diagnosed common mental disorders (O.R. for a 10% increase in Māori ethnic density: 0.92; 95% C.I.: 0.85–0.99; see Table 2).

An increase in area deprivation was consistently associated with increased odds of reporting poor self-rated health and increased psychological distress. For example, as compared to Māori living in the least deprived areas (Quintile 1), Māori living in the most deprived quintile had two times the odds of reporting poor self-rated health (O.R.: 2.04; 95% C.I.: 1.20–3.46; see Table 2, Model 3).

### Māori ethnic density and experienced racial discrimination

In the crude models, a 10% increase in Māori ethnic density was associated with decreased odds of any racial discrimination (Model 1, Table 3). These associations strengthened in Model 2 after adjusting for area deprivation, and in the case of reports of any unfair treatment and any personal attack, became statistically significant. Upon adjustment for individual-level socioeconomic deprivation in Model 3 associations between increased Māori ethnic density and reduced exposure to racial discrimination remained constant.

Similar to the associations found among health outcomes, an increase in area deprivation was strongly associated with increased reports of racial discrimination. Māori living in the fourth and fifth most deprived quintiles experienced increased odds ratios of reporting any personal attack and any racial discrimination, as compared to Māori living in the least deprived quintiles (see Table 3). Effect sizes were largest for reports of any unfair treatment, with Māori living in the most deprived areas experiencing more than twice the odds of reporting any unfair treatment, as compared to Māori living in the least deprived quintile (O.R.: 2.18; 95% C.I.: 1.30–3.63; Table 3, Model 3).

Further analyses conducted to examine the buffering effect of ethnic density on the detrimental association between experiences of racial discrimination and health indicated a tendency for a weaker association between racial discrimination and poor self-rated health, doctor-diagnosed common mental disorders and psychological distress as Māori ethnic density increased, but none of the interaction terms between ethnic density and experienced racial discrimination reached statistical significance.

## Discussion

This is the first study to empirically consider ethnic density effects on health and experiences of racial discrimination in New Zealand, and among indigenous populations globally. We aimed to examine the direct association between ethnic density and health, as well as to explore the main mechanism by which ethnic density impacts on health: a decrease in the experiences of racial discrimination. Although results from the crude models showed either no relationship or a detrimental relationship between ethnic density and health and racism outcomes, these trends were reversed and became statistically significant after introducing area-level deprivation. Fully-adjusted models show that increased Māori ethnic density is associated with decreased reports of poor/fair self-rated health and doctor-diagnosed common mental disorders, as well as with decreased reports of experienced unfair treatment, personal attack, and any racial discrimination.

Importantly, we found that area deprivation masked the protective effects of ethnic density whereby, on adjustment for area deprivation, the association between ethnic density and improved health or reduced racial discrimination strengthened and reached statistical significance. In the instances where this association appeared to be detrimental to health in the crude analysis, adjusting for area deprivation changed the direction of the association into a health-promoting effect. In other words, we found that whereas ethnic density is protective of the health and exposure to racial discrimination of Māori, this effect is concealed by the detrimental effect of area deprivation.

It is now well-established that area deprivation is associated with increased mortality and morbidity, independent of individual-level socioeconomic attributes (Pickett & Pearl, 2001; Riva et al.., 2007), and in the present study we found area deprivation to be independently associated with both poor health outcomes and reported racial discrimination. In the New Zealand context, this means that there is an effect of area deprivation on health and self-reported racial discrimination for Māori over and above individual socioeconomic position. This is an important finding given the over-representation of Māori in areas of high deprivation. Although one might conclude from these findings that contextual effects are particularly relevant for Māori, it was not the aim of our work to differentiate and isolate the independent effects of individual and area-level attributes on health and racial discrimination, but to highlight the detriment caused by area deprivation and its suppressing effect on ethnic density. In fact, we would argue that the dichotomy between compositional and contextual effects commonly discussed in the neighbourhood effects literature does not adequately consider the dynamic relationship between the two and cannot necessarily be directly translated into the Māori context, particularly given potentially specific drivers such as indigenous relationships with the land. Instead, we interpret our findings based on a relational perspective of space and place, where focus is placed on the interaction between the environment and the individual, recognising the mutually reinforcing and reciprocal relationship between people and place (Cummins, Curtis, Diez Roux, & Macintyre, 2007; Curtis & Riva, 2010).

Land continues to be one source of cultural and political identity for Māori (Hitchcock, 2008, pp. 217–243; Kidman, 2012). Relationships with place and patterns of movement between places for indigenous peoples may also be driven by the need to maintain connection with traditional lands and with cultural resources (King et al., 2009). Access to social support structures and resources are potential mechanisms by which ethnic density may be protective for Māori.

However, the spatial distribution of Māori in contemporary New Zealand society is closely linked to colonising processes of land alienation and dispossession, including initial insults of confiscation and resettlement, as well as later periods of urbanisation (Hill, 2012), which have tangible and ongoing detrimental effects on Māori land access and health (Reid & Cram, 2005). As Durie notes, “Loss of land had more than economic implications. Personal and tribal identity were inextricably linked to Papatuanuku – the mother earth – and alienation from land carried with it a serve psychological toll, quite apart from loss of income and livelihood” (Durie, 1997: 33). Urbanisation represented movement away from “tribal centres, the political and social domains of a *Māori* world-view” (Smith, 1996: 347). Policies of ‘pepper potting’ were designed to restrict the concentration of Māori in particular neighbourhoods (Waldergrave, King, Walker, & Fitzgerald, 2006), further undermining Māori social structures. However, Māori also (re)established social institutions and built community in urban contexts (Hill, 2012; Moeke-Pickering, 1996; Walker, 1977).

In the international literature, the spatial differentiation and distribution of indigenous peoples, ethnic minority groups, and majority ethnic groups across neighbourhoods has been referred to as a social manifestation of individual prejudices and institutional discrimination, and as one of the mechanisms by which racism operates (Acevedo-Garcia, 2000; Krieger, 2000). Processes shaping residential segregation include inwardness caused by deprivation and inequality, distrust and fear caused by racism, and the experience of continuous discrimination and social exclusion along ethnic lines (Amin, 2002). In this way, discrimination in housing markets has been found to limit the urban space that members of certain ethnic groups can occupy (Acevedo-Garcia, 2000). While our study shows that ethnic density effects are associated with improved health and decreased experiences of racial discrimination (after adjusting for area deprivation and individual-level socioeconomic position), it also emphasises that the benefits of ethnic density must be interpreted within the current socio-political context. This includes the institutional structures and racist practices that created existing health and socioeconomic inequities in the first place, and that currently maintain the unequal distribution of deprivation by ethnicity. Adequately redressing Māori losses, and addressing poverty and the inequitable distribution of socioeconomic resources by ethnicity and place in New Zealand is vital to improving health and reducing inequalities. Given the racialised nature of access to goods, services, and opportunities within our society, this also requires a strong commitment to eliminating racism. Policies and interventions that mitigate the relationships between deprivation and health and promote healthy, sustainable communities and environments are important. However, commitment and action to the goal of eliminating racism will allow the benefits potentially flowing from strong communities to be fully realised.

Important caveats of this study must be considered. Given the cross-sectional nature of the NZHS, it is not possible to assess the direction of causality. Residual confounding by individual characteristics remains a possibility, as it is likely that there are additional unmeasured aspects of individual and area-level characteristics that we were not able to account for, particularly regarding doctor-diagnosed outcomes and access to care. The self-report measures of health and racism used in this study suffer from cognitive and other psychometric limitations (Krieger, 2000). However, measures such as these have been used in a number of studies of racial discrimination and health (Bécares et al., 2009; Das-Munshi et al., 2010; Halpern & Nazroo, 2000; Harris et al., 2006a, 2006b).

Given confidentiality constraints and limitations of secondary data analysis, we were not able to identify specific drivers of Māori ethnic density. For example, while the ethnic density measures provide information about the concentration of Māori in particular areas, we do not know the specific area where they are living, why they are living there, or if it is the place that they originate from. Future studies incorporating detailed information on the genealogical relationship to place would greatly advance our understanding of ethnic density effects among Māori and other indigenous peoples and inform more specific interventions.

An important strength of this study is the use of data from a nationally representative survey of NZ, which was undertaken close to the 2006 population census (to which data was geocoded), avoiding problems of temporality, which are common in other studies of ethnic density.

## Conclusion

This study documents, for the first time, protective ethnic density effects on the health of Māori and on their exposure to racial discrimination. Findings of this study also demonstrate that this protective ethnic density effect is masked by the concomitant association between areas of high Māori density and areas of high deprivation.

## Figures and Tables

**Table 1:** Descriptive characteristics of the 2006/07 New Zealand Health Survey Māori sample.

	Māori (*n* = 3160)
	Weighted % (unweighted *n*)
Markers of health	
Fair/poor self-rated health	13 (449)
Doctor-diagnosed common mental disorder	14 (485)
Psychological Distress (Kessler-10), *M*(SD)	4.6 (5.6)
Racism and discrimination	
Any personal attack (verbal or physical)	24 (783)
Any unfair treatment (by a health professional, at work, or gaining housing)	13 (461)
Overall discrimination	29 (977)
Ethnic density	
Māori ethnic density, *M*(SD) [range]	23.4 (17.2) [2–86]
Area deprivation (NZDep06)	
1. Least deprived	9 (245)
2	10 (297)
3	16 (472)
4	25 (761)
5. Most deprived	40 (1385)
Sex	
Female	54 (1955)
Male	46 (1205)
Age, *M*(SD)	37 (16)
Education	
No school	31 (1008)
Secondary school qualification	27 (771)
Post-secondary school qualification	42 (1374)
Work status	
Working in paid employment	61 (1808)
Not in paid employment, looking for job	10 (300)
Not in paid employment, not looking for a job	28 (1040)
Equivalised household income, *M*(SD) [Median]	$45,535 ($30,931) [$37,415]

Estimates weighted to account for non-response of eligible participants and the unequal probability of being sampled.

**Table 2:** Association between a 10% increase in Māori ethnic density and poor self-rated health, doctor-diagnosed common mental disorders, and psychological distress (Kessler-10).

	Fair/poor self-rated health			Doctor-diagnosed common mental disorder			Psychological distress		
	Model 1	Model 2	Model 3	Model 1	Model 2	Model 3	Model 1	Model 2	Model 3
	O.R. (95% C.I.)	O.R. (95% C.I.)	O.R. (95% C.I.)	O.R. (95% C.I.)	O.R. (95% C.I.)	O.R. (95% C.I.)	Coeff (S.E.)	Coeff (S.E.)	Coeff (S.E.)
**Māori ethnic density**	1.01 (0.96–1.07)	0.92 (0.85–0.99)	0.91 (0.84–0.98)	0.95 (0.90–1.01)	0.93 (0.86–1.00)	0.92 (0.85–0.99)	0.09 (0.06)	−0.09 (0.08)	−0.13 (0.08)
**Area deprivation**									
1. Least deprived		1.00	1.00		1.00	1.00		0.00	0.00
2		1.57 (0.90–2.74)	1.46 (0.83–2.56)		1.30 (0.81–2.09)	1.19 (0.74–1.92)		0.64 (0.51)	0.40 (0.50)
3		1.69 (1.01–2.83)	1.47 (0.87–2.48)		1.10 (0.70–1.72)	0.91 (0.58–1.44)		0.99 (0.47)	0.57 (0.46)
4		1.83 (1.12–3.01)	1.61 (0.97–2.65)		1.39 (0.91–2.12)	1.14 (0.74–1.75)		1.50 (0.45)	1.05 (0.44)
5. Most deprived		2.56 (1.52–4.30)	2.04 (1.20–3.46)		1.37 (0.87–2.16)	1.00 (0.63–1.60)		1.76 (0.49)	1.03 (0.48)

Model 1 adjusts for age and sex; Model 2 adjusts for age, sex, and area deprivation; Model 3 adjusts for age, sex, area deprivation, education, work status, and equivalised household income.

**Table 3:** Association between a 10% increase in Māori ethnic density and experienced unfair treatment, any personal attack, and any racial discrimination.

	Any unfair treatment			Any personal attack			Any racial discrimination		
	Model 1	Model 2	Model 3	Model 1	Model 2	Model 3	Model 1	Model 2	Model 3
	O.R. (95% C.I.)	O.R. (95% C.I.)	O.R. (95% C.I.)	O.R. (95% C.I.)	O.R. (95% C.I.)	O.R. (95% C.I.)	O.R. (95% C.I.)	O.R. (95% C.I.)	O.R. (95% C.I.)
**Māori ethnic density**	0.97 (0.92–1.03)	0.90 (0.83–0.97)	0.90 (0.83–0.98)	0.95 (0.90–1.00)	0.91 (0.85–0.97)	0.92 (0.86–0.98)	0.95 (0.91–0.99)	0.90 (0.84–0.96)	0.90 (0.85–0.96)
**Area deprivation**									
1. Least deprived		1.00	1.00		1.00	1.00		1.00	1.00
2		1.45 (0.84–2.47)	1.39 (0.81–2.39)		1.26 (0.83–1.91)	1.28 (0.85–1.94)		1.21 (0.81–1.80)	1.21 (0.81–1.80)
3		1.49 (0.91–2.46)	1.42 (0.86–2.34)		1.28 (0.87–1.87)	1.30 (0.89–1.91)		1.44 (1.00–2.07)	1.43 (0.99–2.07)
4		2.13 (1.33–3.41)	2.03 (1.26–3.27)		1.40 (0.97–2.02)	1.46 (1.01–2.12)		1.77 (1.25–2.51)	1.78 (1.25–2.54)
5. Most deprived		2.26 (1.36–3.74)	2.18 (1.30–3.63)		1.55 (1.04–2.31)	1.70 (1.13–2.55)		1.81 (1.23–2.64)	1.88 (1.28–2.77)

Model 1 adjusts for age and sex; Model 2 adjusts for age, sex, and area deprivation; Model 3 adjusts for age, sex, area deprivation, education, work status, and equivalised household income.
